# Micromechanical study of the load transfer in a polycaprolactone–collagen hybrid scaffold when subjected to unconfined and confined compression

**DOI:** 10.1007/s10237-017-0976-5

**Published:** 2017-11-11

**Authors:** A. P. G. Castro, D. Lacroix

**Affiliations:** Department of Mechanical Engineering, INSIGNEO Institute for in Silico Medicine, University of Sheffield, Pam Liversidge Building, Mappin Street, Sheffield, S1 3JD UK

**Keywords:** Tissue engineering, Collagen, Polycaprolactone, Finite element simulation, Biomechanical stimuli

## Abstract

Scaffolds are used in diverse tissue engineering applications as hosts for cell proliferation and extracellular matrix formation. One of the most used tissue engineering materials is collagen, which is well known to be a natural biomaterial, also frequently used as cell substrate, given its natural abundance and intrinsic biocompatibility. This study aims to evaluate how the macroscopic biomechanical stimuli applied on a construct made of polycaprolactone scaffold embedded in a collagen substrate translate into microscopic stimuli at the cell level. Eight poro-hyperelastic finite element models of 3D printed hybrid scaffolds from the same batch were created, along with an equivalent model of the idealized geometry of that scaffold. When applying an 8% confined compression at the macroscopic level, local fluid flow of up to 20 $\upmu$m/s and octahedral strain levels mostly under 20% were calculated in the collagen substrate. Conversely unconfined compression induced fluid flow of up to 10 $\upmu$m/s and octahedral strain from 10 to 35%. No relevant differences were found amongst the scaffold-specific models. Following the mechanoregulation theory based on Prendergast et al. (J Biomech 30:539–548, 1997. 10.1016/S0021-9290(96)00140-6), those results suggest that mainly cartilage or fibrous tissue formation would be expected to occur under unconfined or confined compression, respectively. This in silico study helps to quantify the microscopic stimuli that are present within the collagen substrate and that will affect cell response under in vitro bioreactor mechanical stimulation or even after implantation.

## Introduction

The introduction and rapid expansion of additive manufacturing techniques applied to the tissue engineering (TE) field have led to virtually unlimited scaffold configurations for a large number of applications (Willie et al. 2010; Mercado-Pagán et al. 2015; Hollister et al. 2016; Neves et al. 2016). Although various optimization methods have been proposed to identify the most appropriate scaffold geometry or mechanical properties, most studies rely on a trial-and-error approach (Dias et al. 2014; Campos Marin and Lacroix 2015; Weisgerber et al. 2016). In silico studies are now fundamental to improve and tailor new TE applications and strategies (Stylianopoulos and Barocas 2007; Vanegas-Acosta et al. 2011; Xu et al. 2013; Manzano et al. 2014; Campos Marin and Lacroix 2015). One of the key solutions is to combine numerical and experimental results, supporting and providing retro-feedback to the advancements on in vivo, ex vivo, in vitro and in silico studies (Prendergast et al. 2010; Freutel et al. 2014; Viceconti 2015). Numerical simulation has been reported as a viable approach to evaluate the performance of TE systems (Porter et al. 2005; Byrne et al. 2011; Sandino and Lacroix 2011; Zhao et al. 2015; Boccaccio et al. 2016). It can provide a valuable insight on the adjacent tissues and cells response to loading or other biomechanical and biochemical stimuli (Lacroix and Prendergast 2002; Sandino and Lacroix 2011; Carlier et al. 2014), leading to geometry, function or material optimization (Chen et al. 2011; Papantoniou et al. 2014; Campos Marin and Lacroix 2015; Boccaccio et al. 2016; Rahbari et al. 2016).

The mechanobiological behaviour occurring at the tissue level of TE constructs can be evaluated from the mechanoregulatory pathway theory of Prendergast et al. (1997) (later modified in the works of Lacroix and Prendergast 2002 and Olivares et al. 2009). The major focus in this study is on the transmission of mechanical stimuli within a hybrid TE construct composed of a stiff scaffold made of PCL to the local environment of a soft collagen substrate. Collagen is well known to be a hyper-poro-viscoelastic natural biomaterial, given to its abundance and intrinsic biocompatibility, and is one of the most frequent choices in TE applications (Weisgerber et al. 2016). The ground concept is that one shall be able to extrapolate the potential cell behaviour in terms of alignment, migration, proliferation or differentiation by predicting shear strain and fluid flow in the substrate layers adjacent to the scaffold (Lacroix and Prendergast 2002; Sandino and Lacroix 2011; Zhao et al. 2015). Unconfined and confined compression of such constructs not only simulates common bioreactor environment, but also links with the in vivo constrains of such a scaffold being implanted on a long bone (such as femur, tibia or humerus) (Sanz-Herrera et al. 2009; Willie et al. 2010).

Previous works on mechanobiology-related numerical simulation of TE scaffolds used FE, computational fluid dynamics (CFD) or fluid–structure interaction (FSI) methods on geometrically ideal scaffold models (Stops et al. 2008; Olivares et al. 2009; Zhao et al. 2015; Boccaccio et al. 2016; Seifer and Wagner 2016) or scanned-image-based reconstructed models of single scaffold samples (Sandino et al. 2008; Milan et al. 2009; D’Amore et al. 2014). Sandino and Lacroix (2011) modelled the effect of macroscopic compressive strain and dynamic fluid flow in a calcium–phosphate-based glass porous scaffold to predict how local fluid velocities would influence cell differentiation. Porter et al. (2005) created CFD models to evaluate fluid shear stress and its association with cell proliferation and bone gene markers, depending on the flow rate and scaffold microarchitecture. Zhao et al. (2015) developed a FSI study to achieve a multiscale and multiphysics approach for the modelling of the mechanical stimulation of cells, compression and perfusion stimuli were simulated to evaluate osteoblasts response in a poly(d,l-lactide) scaffold, and the controlled combination of both stimuli was confirmed as favourable for osteogenic differentiation.

Consequently, studies in this field were able to evaluate strain and fluid conditions under compression and/or perfusion of the scaffolds. They also demonstrated the validity and robustness of numerical models for predicting scaffold integration within the host tissue or even the optimized conditions for bone regeneration (Sandino and Lacroix 2011; Zhao et al. 2015; Boccaccio et al. 2016; Guyot et al. 2016; Seifer and Wagner 2016). Multiscale poro-hyperelastic finite element (FE) models allow multi-level insight, from the macroscopic compression of the construct to the microscopic level of potential substrate–cell interaction, thus characterizing the cell host environment (Carlier et al. 2014; Deponti et al. 2014). However, to the authors’ best knowledge, none of the mechanobiological studies in the literature account for potential scaffold variability or cell substrate behaviour.

The scaffold model under evaluation is the commercially available bone TE scaffold 3D Insert®PCL (3D Biotek, USA). It has been reported that such scaffolds may present some variability, in terms of overall dimensions and porosity. This variability of the scaffolds, motivated by the manufacturing processes, may potentially affect both substrate and cellular environment under a TE construct (Dias et al. 2014; Campos Marin and Lacroix 2015; Brunelli et al. 2017).

Therefore, the main goal for this study was to test how moderate compressive loading would potentially affect the mechanoregulatory pathways in tissue differentiation, namely in what concerns to microscopic and macroscopic stress–strain conditions and also fluid flow velocities (Prendergast et al. 1997; Sarkar et al. 2006; Sandino and Lacroix 2011; Zhao et al. 2015; Guyot et al. 2016; Offeddu et al. 2016; Wittkowske et al. 2016). The main hypotheses for this study are thus to assess whether geometry differences at the macro- and microscopic level (of the scaffolds) would cause different stimuli for tissue formation and also if the compression mode (unconfined or confined) would alter significantly the overall biomechanical behaviour of the scaffold–substrate construct.

**Fig. 1:**
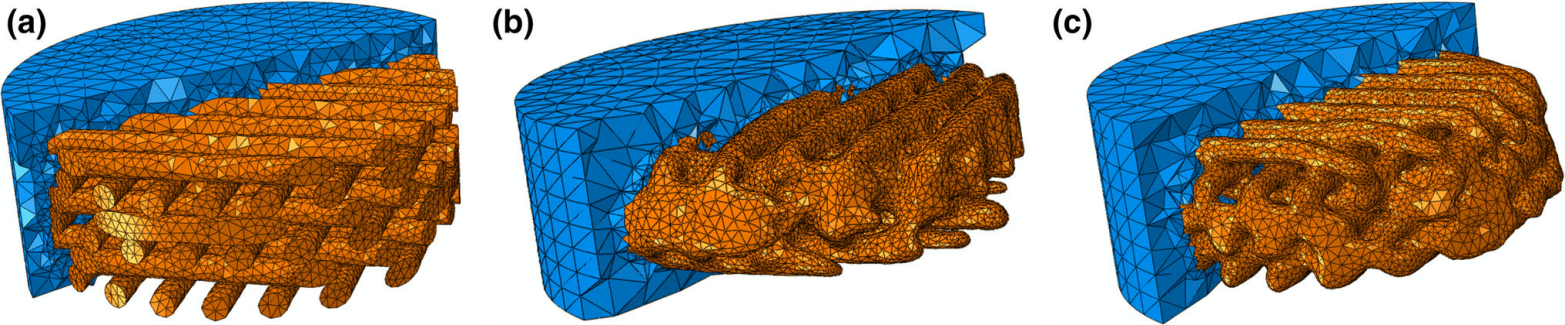
Section cut of PCL scaffold and collagen substrate construct (orange and blue, respectively): **a** CAD model, **b** and **c** two reconstructed micro-CT samples

**Table 1:** Height, porosity and total number of elements and nodes of the nine construct FE models

Model	Total height (mm)	PCL scaffold porosity (%)	Total number of elements	Total number of nodes
CAD model	2.35	46.0	206,945	279,548
$\mu CT \#1$	2.06	40.6	213,639	293,228
$\mu CT \#2$	2.22	41.3	244,090	334,867
$\mu CT \#3$	2.09	45.8	240,414	329,882
$\mu CT \#4$	2.16	46.3	246,378	337,807
$\mu CT \#5$	2.13	42.5	232,513	319,151
$\mu CT \#6$	2.15	44.9	244,506	335,272
$\mu CT \#7$	2.20	42.1	235,913	323,745
$\mu CT \#8$	2.17	45.8	238,102	326,726

**Table 2:** Poro-hyperelastic material parameters for the components of the construct

Material	$K_0^*(m^{4}/Ns)$	M	Void ratio	${C}_{10}$ (kPa)	${D}_{1}$ (kPa)
PCL (Brunelli et al. 2017)	$1.00 \times 10^{-14}$	–	0.786	4.17	0.0144
Collagen (Castro et al. 2016)	$1.70 \times 10^{-10}$	1.8	499	0.0131	2312

$K_0^*$ is the zero-strain hydraulic permeability; *M* is a dimensionless nonlinear permeability parameter; void ratio is a quantity related to the porosity of a given material; ${C}_{10}$ and ${D}_{1}$ are stiffness-related parameters for the Neo-Hookean hyperelasticity model

In other words, the biomechanical performance of a scaffold–substrate construct based on the ideal 3D Insert PCL scaffold model is compared with eight constructs based on the reconstructed models (after micro-CT scanning of eight commercially available units of this 3D Insert PCL) under FE simulation of unconfined and confined bioreactor environment, so that it can be assessed how the alterations to the testing setup and materials at the macro- and microscopic level (of the scaffolds) are due to cause different stimuli for tissue formation.

## Materials and methods

TE constructs composed by 3D Insert PCL scaffolds (3D Biotek, USA) and highly hydrated collagen hydrogels (0.20% collagen concentration by weight) were modelled. The 3D Insert PCL scaffold is evaluated in two variants: (one) computer-aided design (CAD) model and (eight) micro-computed tomography ($\mu$CT) scanned models of manufactured samples (Fig. 1).

The CAD model represents the ideal geometry of this scaffold model and was previously presented by Campos Marin and Lacroix (2015). It consists of regularly distributed fibres with a diameter and inter-fibre spacing of $300\,\upmu$m. There are six layers of fibres, with layer to layer offset of $90^{\circ }$ in the orientation of the fibres. Overall diameter is 5 mm.

The eight scaffolds samples were scanned at 40 kV, 10 W, and 250 mA, using SkyScan 1172®(Bruker, Belgium), with a voxel size of 17.4 $\upmu$m. During the scanning, scaffolds were automatically rotated and consecutive projection images were acquired by the detector. The software used for image reconstruction was ScanIP (Simpleware Ltd, UK) (Brunelli et al. 2017), which has been thoroughly used to the generation of a FE mesh from micro-CT data and thus ensures the minimization of potential errors resulting from this process (Guldberg et al. 1998; Young et al. 2008). The FE software used for modelling and simulation was Abaqus (Dassault Systèmes, France), and post-processing was done with Paraview (Kitware, USA).

PCL and collagen were modelled as poro-hyperelastic, using van der Voet strain-dependent permeability model for the collagen substrate and Neo-Hookean formulation for the ground substances of both materials. The numerical modelling of highly hydrated collagen hydrogels as poro-hyperelastic materials has already been extensively described in Castro et al. (2016). Quadratic ten-node tetrahedral elements (C3D10MPH) were used, in order to bear with poro-elasticity and large deformation requirements. No contact was defined between the PCL and collagen, assuming that the collagen would attach well to the PCL. Congruent mesh was used. Table 1 summarizes the properties of each FE model. (Mesh dimensions were optimized after a mesh convergence study.) Table 2 displays the material properties, extracted from in-house experiments.

Unconfined and confined uniaxial compression was imposed on the top of the construct (8% ramp compression during 10 s and then hold for 290 s). 8% compression is believed to be enough to induce a response at the cellular level (Bandeiras et al. 2015; Seifer and Wagner 2016). It must be highlighted that beyond 8% compression, these scaffolds start showing signs of plastic deformation; hence, this was the maximum deformation chosen (Brunelli et al. 2017). The bottom layer of the construct was fully constrained at all times, and fluid exudation was allowed through the free surfaces (i.e., lateral and top surfaces on the unconfined mode; only the top surface on the confined mode), in order to simulate common bioreactor environments.

**Fig. 2:**
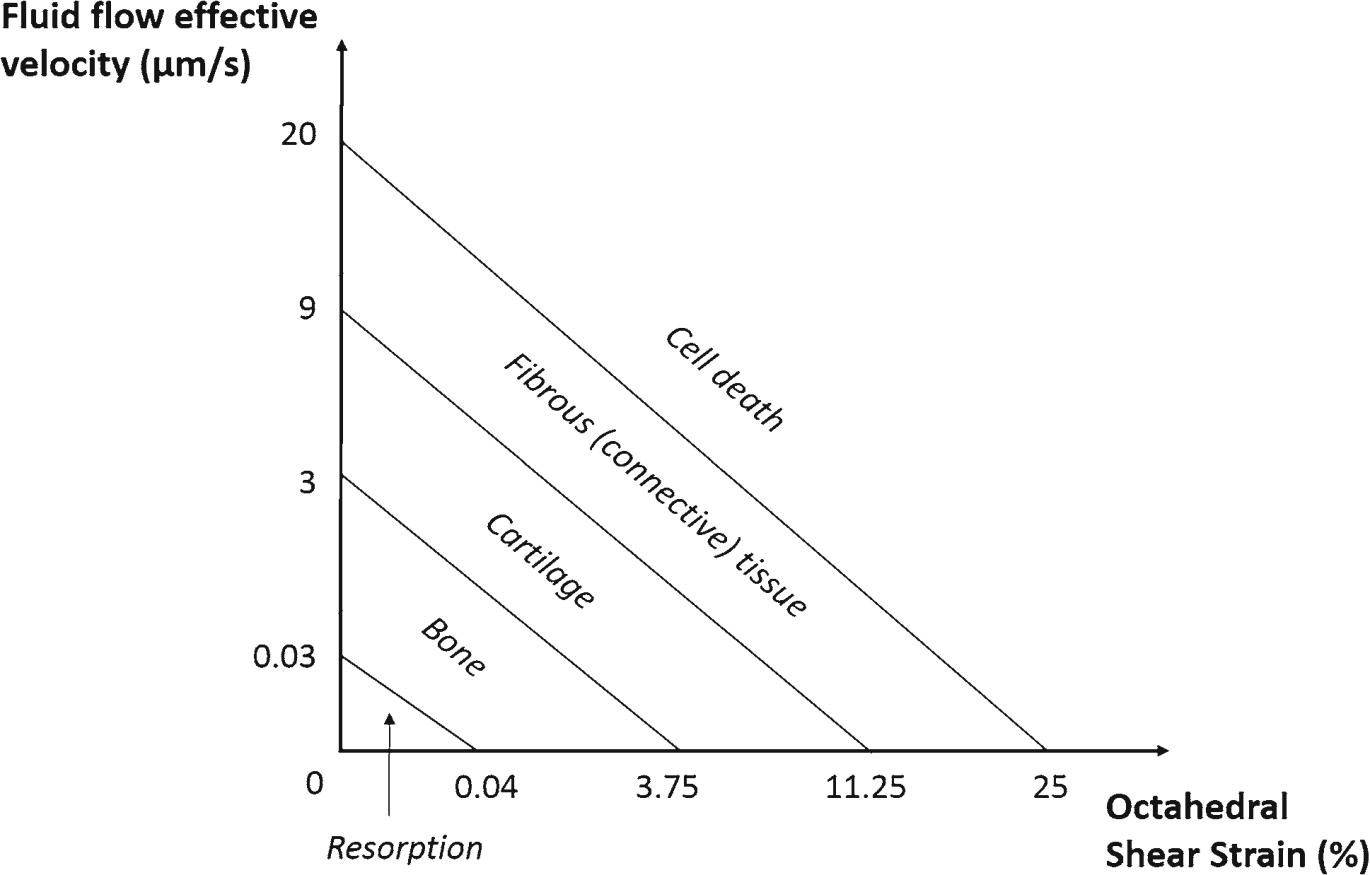
Mechanoregulatory pathway diagram. Adapted from Lacroix and Prendergast (2002)

Effective stress (ES, calculated over the longitudinal axis) is extracted from the bottom layer of the collagen substrate, throughout the duration of the confined compression test, in order to do a preliminary evaluation of the macroscale behaviour of the nine FE models. Fluid flow effective velocity (FLVEL) and octahedral shear strain (OSS) were calculated for both scaffold and substrate components, at the peak stress instant (at 10 s), for the nine FE models and two compression modes. The last output variable is denominated “mechanobiological output” (MBO). This results from a combination of FLVEL and OSS variables in accordance with the mechanoregulatory pathway theory of (Prendergast et al. 1997), i.e., depending on FLVEL and OSS levels, cells attached in the substrate will potentially differentiate onto osteoblasts, chondrocytes or fibroblasts (bone, cartilage or fibrous tissue, respectively). Resorption and cell death are the lower and upper extremes, meaning insufficient stimulation or over-stimulation of the cells, respectively (Lacroix and Prendergast 2002; Sandino and Lacroix 2011). The respective diagram is shown in Fig. 2. It must be highlighted that due to the short-term nature of this test, which aims to identify the direct response of the hydrogel to the mechanical stimuli and how that response would be transmitted to the attached cells, no tissue adaptation or alteration along time was considered.

## Results

**Fig. 3:**
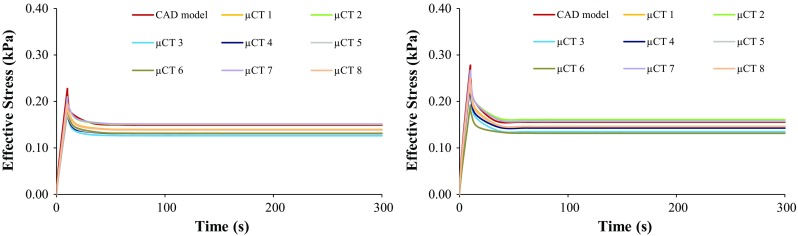
Effective stress over time on the collagen layer under 8% unconfined (left) and confined (right) compression, for the nine FE models

Figure 3 displays the comparison between the ES calculated for the nine construct FE models, under 8% unconfined and confined compression. Under unconfined compression, the peak stress calculated with the CAD model was 15% higher than the average of the micro-CT models. This difference dropped to 8% at the end of the relaxation. The stress reduction from peak to relaxation stage was 35% on the CAD model, while the average of the micro-CT models was 29%. Under confined compression, the peak stress calculated with the CAD model was 10% higher than the average of the micro-CT models, reducing to 6% at the end of the test. The stress reduction from peak to relaxation stage was 44% on the CAD model, while the average of the micro-CT models was 41%.

**Fig. 4:**
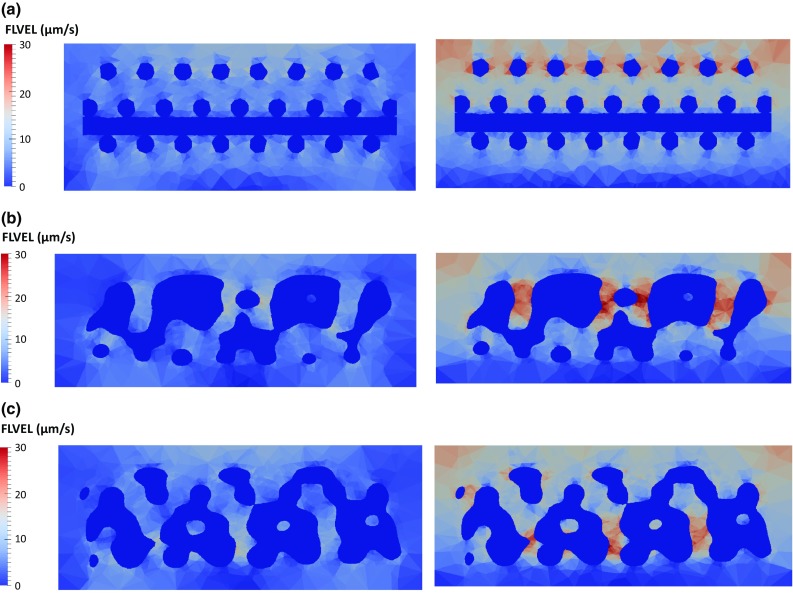
Distribution of FLVEL over a section cut of the construct under 8% unconfined (left) and confined (right) compression ($t=10$ s): **a** CAD model, **b** micro-CT model #1, **c** micro-CT model #2

**Fig. 5:**
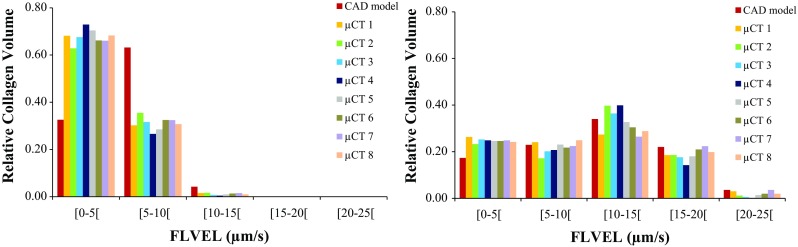
Distribution of FLVEL ($\upmu$m/s) in the collagen elements under 8% unconfined (left) and confined (right) compression against relative collagen volume ($t=10$ s), for the nine FE models

Figure 4 shows the distribution of FLVEL over an axial section cut of the constructs at peak stress instant, under 8% unconfined and confined compression, respectively. Figure 5 shows the distribution of FLVEL throughout the volume of the collagen substrate, also under unconfined and confined compression. FLVEL is negligible on the bottom layers of the collagen substrate, for the confined case. For this case, FLVEL is almost evenly distributed within the inner layers of collagen substrate, where it ranges between 5 and 15 $\upmu \hbox {m}/\hbox {s}$ in the majority of the visible area (average 56% of the collagen elements in this range), for both CAD and micro-CT-derived models. Some fluid concentration spots, noted by FLVEL higher than average, are observed on the collagen around the scaffold fibres, particularly in the more irregular areas of the micro-CT-derived construct models. Nevertheless, 98% of the collagen elements present FLVEL levels not higher than $20\,\upmu \hbox {m/s}$. When the compression is unconfined, internal FLVEL goes up to $10\,\upmu \hbox {m/s}$, with lower magnitudes on the lateral boundaries (average 64% of the collagen elements under $5\,\upmu \hbox {m/s}$). It is worth noticing that the CAD model presents a different tendency from the micro-CT-derived models, by presenting 33% of the collagen elements up to $5\,\upmu \hbox {m/s}$ and 63% between 5 and $10\,\upmu \hbox {m/s}$. Fluid concentration spots are again noticed near the scaffold fibres for the nine models.

**Fig. 6:**
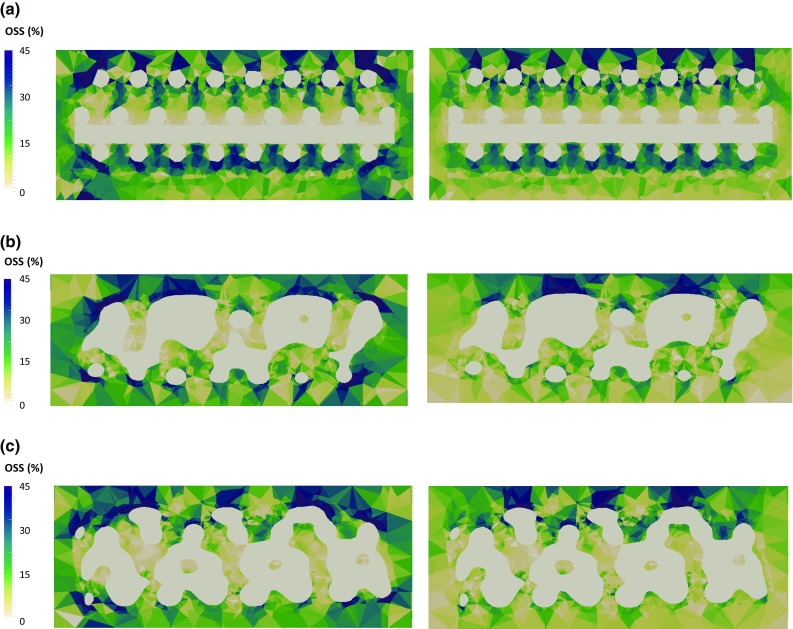
Distribution of OSS over a section cut of the collagen substrate under 8% unconfined (left) and confined (right) compression ($t=10$ s): **a** CAD model, **b** micro-CT model #1, **c** micro-CT model #2

**Fig. 7:**
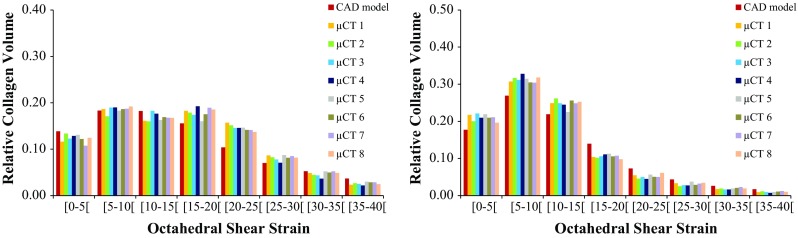
Distribution of OSS within the collagen elements under 8% unconfined (left) and confined (right) compression against relative collagen volume ($t=10$ s), for the nine FE models

Figure 6 displays the distribution of OSS over the same section cut of the collagen substrate at peak stress instant, under unconfined and confined compression, respectively. Figure 7 shows the distribution of OSS throughout the volume of the collagen substrate. When the compression is unconfined, OSS is mostly distributed from 10 to 35% (average 80% of the collagen elements in this range), with lower magnitudes on the internal collagen surfaces that are contacting the scaffold. Strain concentration spots are visible near the top layers of the substrate (up to 5% of the elements above 40% of OSS). Under confined compression, OSS goes up to 20% in the majority of the collagen elements (average 87%), reaching higher values in the top layers of this substrate (but only 1% of the elements above 40%).

**Fig. 8:**
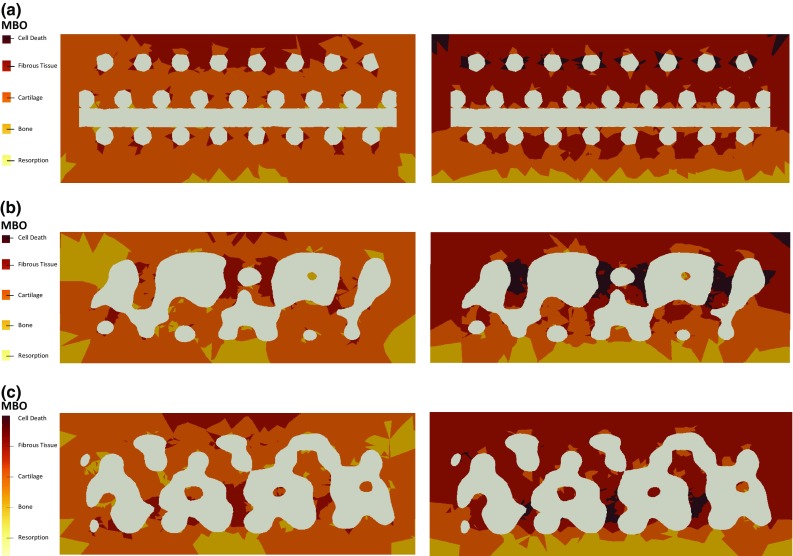
Distribution of MBO over a section cut of the collagen substrate under 8% unconfined (left) and confined (right) compression ($t=10$ s): **a** CAD model, **b** micro-CT model #1, **c** micro-CT model #2

**Fig. 9:**
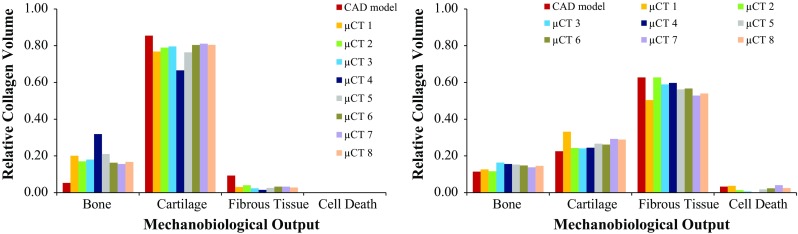
Distribution of MBO within the collagen elements under 8% unconfined (left) and confined (right) compression against relative collagen volume ($t=10$ s), for the nine FE models

Figure 8 plots MBO distribution over the same section cut of the constructs at peak stress instant, under unconfined and confined compression, respectively. Figure 9 displays the MBO distributions of the nine FE models, accordingly to the compression mode. It can be observed that the nine FE models present equivalent outcomes throughout both compression modes. For the unconfined case, distribution of MBO is even on the CAD model, while the micro-CT-derived models show some areas with varied outcomes, promoting either bone or fibrous tissue formation. Nevertheless, cartilage is promoted in the majority of the elements (78%), with small percentage of bone (18%) and even smaller of fibrous tissue (4%). For the confined case, the bottom layers and the areas closer to the scaffold fibres would mostly promote the formation of cartilage or even bone tissue. For the micro-CT-derived models in particular, some internal areas where cell death could happen are also visible. Fibrous tissue will potentially prevail over the other tissues (57%, against 27% for cartilage and 14% for bone). It must be highlighted that a small probability of cell death occurs in this case (2% of the volume).

## Discussion

At the macromechanical level, the difference between the ES calculated for the collagen substrate around the CAD model and the eight micro-CT samples was not larger than 21% (at the peak stress, under unconfined compression, whereas the maximum difference for confined compression was 16%), suggesting that the overall behaviour of the construct would still obey to comparable macroscopic deformation patterns under the 8% uniaxial unconfined and confined compression (materials-wise). No direct link was found between the different height of the constructs or scaffold porosity and the calculated ES, FLVEL and OSS, which seem to be consistent along the nine FE models. The only exception is for the FLVEL distribution on the collagen substrate of the CAD model under unconfined compression, which is inverse from micro-CT-derived models, but that does not change the overall outcomes (Fig. 5).

At the micromechanical level, the different geometries of the scaffolds seem to be inducing local higher deformation spots, as well as different patterns for fluid exudation. This study shows that 8% confined compression of the construct can prompt relatively high velocities for the fluid flow across the collagen substrate (up to $20\,\upmu \hbox {m/s}$ in 98% of the elements, averaging between 10 and $15\,\upmu \hbox {m/s}$). In accordance with expected consolidation behaviour of tissues (Chegini and Ferguson 2010; Offeddu et al. 2016), FLVEL magnitude is observed to be higher in the top collagen layers (during the initial compression) and close to null in the bottom layers. When the compression is unconfined, FLVEL magnitudes drop to half, showing that the lateral confinement plays an important role in the way fluid flows through the construct.

While previous numerical studies have focused on fluid perfusion through the scaffold (Sandino and Lacroix 2011; Zhao et al. 2015) or on the deformation of the scaffold itself (Milan et al. 2009) to evaluate potential cell differentiation, the current outcomes seem to indicate that 8% compression at the construct level would be able to induce relatively high OSS magnitudes within this collagen substrate (Prendergast et al. 1997; Milan et al. 2009, 2010; Byrne et al. 2011; Sandino and Lacroix 2011). OSS levels are higher on the unconfined compression case, which reveals that the absence of the lateral constraint allows for higher shear deformation even with lower FLVEL magnitudes through the collagen substrate. In effect, an average of 52% of the elements in the collagen substrate (across the nine FE models) have shown OSS levels of up to 10%. In addition, 35% of the elements have shown OSS levels between 10 and 20% (Prendergast et al. 1997; Isaksson et al. 2007).

The analysis of the mechanoregulatory outcomes to be expected under 8% uniaxial unconfined compression suggests that the cells attached onto the collagen substrate will mostly differentiate onto chondrocytes, regardless of the location within the substrate. No cell death is expected to occur, along with reduced probability of fibroblasts formation. Under confined compression, the formation of fibroblasts prevails over the formation of osteoblasts and chondrocytes (Prendergast et al. 1997; Lacroix and Prendergast 2002; Isaksson et al. 2007; Sandino and Lacroix 2011). The lower layers of the collagen substrate and the areas closer to the PCL scaffold are subjected to lower stimulation (resulting in lower FLVEL and OSS and thus promoting the formation of osteoblasts and chondrocytes), most likely due to the consolidation behaviour of the soft collagen hydrogel under compression and also due to the proximity to the stiffer scaffold material.

The fluid flow is most likely the key factor for triggering such processes and would also be important to mechanobiological phenomena in adjacent tissues, namely bone healing and adaptation, after scaffold implantation (Lacroix and Prendergast 2002; Byrne et al. 2007; Pereira et al. 2015; Wittkowske et al. 2016). It must be highlighted that the mechanobiological environment at the tissue level is predicted to be similar along the nine different constructs under analysis, i.e., the observed geometrical variability of the scaffolds seems again to not be affecting substantially the overall outcomes regarding the cell substrate. These are very important findings regarding the effectiveness of such TE constructs and also for the validity of the moderate uniaxial compression simulation protocols, particularly in what concerns to the collagen substrate areas directly attached to the PCL scaffold.

It must also be highlighted that the probability of cell death seems to be reduced, under both simulated mechanical conditions. In fact, the irregularities of the micro-CT-derived models seem to be causing areas of higher fluid velocity (on average 2% of the whole collagen substrate area for the confined compression case), namely where the fluid would be trapped and thus not flowing through the construct as happened in the CAD model. These irregularities seem to not be determinant for the behaviour of the overall construct, and they are also less noticed under unconfined compression. Such outcome could also be associated with the area where the compressive load is being applied (valid not only for the micro-CT-derived models, but also for the CAD model) (Prendergast et al. 1997; Porter et al. 2005; Byrne et al. 2011).

Based on these outcomes, one may extrapolate that similar FE simulation protocols would be valid for the optimization of scaffold design or tailoring of biomaterials targeting bone TE strategies, i.e., forthcoming research may focus on finding the optimal combination between geometry and material properties to promote mechanobiological cell stimuli under an environment that mimics post-implantation on bone. The confinement of the construct is playing a major role on MBO, changing the most likely promoted tissue from cartilage to fibrous tissue, probably meaning that restricting the lateral deformation of the construct is inducing an over-stimulation effect. Nevertheless, the probability of bone formation is not significantly altered, but it is under 20% for both cases (in regards to the relative collagen volume), which is relatively low if one considers that these scaffolds are mostly used for bone TE applications. Therefore, lower magnitudes of compressive loads would be needed to target bone regeneration. Nevertheless, bone could also be formed through endochondral ossification later on, but this study is not evaluating such possibility (Isaksson et al. 2007; Carlier et al. 2014).

In fact, the short simulation time, which typifies this as an almost non-dynamic study, is one of the limitations of this work. This study also only looks at the effect of mechanical loading and not at the effect of biochemical stimulation through material–cell interactions or growth factors (Lacroix and Prendergast 2002; Huang et al. 2016). Finally, it must be highlighted here that it is important to prevent the PCL scaffold to go under plastic deformation, which may happen at higher strain levels, and the applied stimuli was found to be appropriate (Bandeiras et al. 2015; Brunelli et al. 2017). Higher loading magnitudes would be important to understand the risk of causing cell death (rather than promoting tissue formation) under more demanding solicitations.

## Conclusions

This FE investigation on the mechanobiological behaviour of PCL scaffold surrounded by collagen substrate for cell attachment under unconfined and confined compression helps demonstrating the importance of FE simulations for the mechanobiological analysis of complex TE constructs and strategies, and even on the production control perspective.

At the macroscopic level, it was found that the geometric variability of the scaffold would induce differences only at the local tissue level, but the broad mechanobiological environment of the tissues where such scaffold would be implanted (primarily bone) would be more affected by the confinement mode than by the geometrical differences amongst the scaffolds here analysed.

At the microscopic level, this work reveals that an 8% confined compression would potentially induce the formation of fibrous tissue from a large part of the cells attached to the collagen substrate. It was demonstrated that such response from the cells is associated with the generated levels of fluid shear strain (Prendergast et al. 1997; Lacroix and Prendergast 2002; Sandino and Lacroix 2011; Bandeiras and Completo 2017). The occurrence of some abnormal higher deformation areas and possible fluid flow trapping spots on the micro-CT-derived models is a concern to be addressed when manufacturing TE devices, as they may cause cell death. Under unconfined compression, the probability of inducing formation of cartilage prevails over bone and fibrous tissue (Prendergast et al. 1997; Lacroix and Prendergast 2002; Sandino and Lacroix 2011).

In conclusion, in silico confined compression introduces an effect of over-stimulation, suggesting that related experimental and numerical studies may be more realistic if performed under unconfined compression. The insight provided in terms of fluid flow and strain distribution inside the construct allowed to conclude that the moderate compression of 8% at a strain rate of $0.008\,\hbox {s}^{-1}$ applied at the macroscopic level of the construct generated relevant microscopic shear strain at the substrate tissue level, which should potentially trigger cell differentiation within this collagen substrate.
